# Social Exclusion Shifts Personal Network Scope

**DOI:** 10.3389/fpsyg.2019.01619

**Published:** 2019-07-24

**Authors:** Joseph B. Bayer, David J. Hauser, Kinari M. Shah, Matthew Brook O’Donnell, Emily B. Falk

**Affiliations:** ^1^School of Communication, The Ohio State University, Columbus, OH, United States; ^2^Communication Studies, University of Michigan, Ann Arbor, MI, United States; ^3^Department of Psychology, Queen’s University, Kingston, ON, Canada; ^4^Department of Psychology, University of Michigan, Ann Arbor, MI, United States; ^5^Annenberg School for Communication, University of Pennsylvania, Philadelphia, PA, United States

**Keywords:** cyberball, ostracism, social network, activation, cognition, word-of-mouth, online, availability

## Abstract

Social exclusion has the potential to alter subsequent social interactions with the members of personal networks, especially given their online availability in contemporary life. Nonetheless, there is minimal research examining how social challenges such as exclusion alter ensuing interactions with personal ties. Here, we tested whether being excluded during a social interaction changed which relationships are most salient in an ostensibly unrelated, online news sharing task. Across three operationalizations of tie strength, exclusion (vs. inclusion) increased sharing to close friends, but (unexpectedly) decreased sharing to close family members. The findings provide preliminary evidence that negative encounters may shift attention toward certain types of network ties and away from others. Future work is needed to examine how social experiences influence personal network scope – i.e., who comes to mind – in the background of daily life.

## Introduction

One challenging event that occurs regularly in daily life is social exclusion, which can increase negative mood (Blackhart et al., 2009) whether it occurs *via* face-to-face, text message, or social media interactions (Smith, 2004; Schneider et al., 2017; Covert and Stefanone, 2018; Hales et al., 2018). Some work also shows that social exclusion can diminish belonging, control, and self-esteem (Gerber and Wheeler, 2009), though the latter effect remains unclear (c.f., Blackhart et al., 2009). Given the mental costs of exclusion, individuals often respond by reaching out to others, consciously or unconsciously. Indeed, past research suggests that people react anti-socially if subsequent inclusion seems unlikely, but otherwise pursue prosocial goals (DeWall and Richman, 2011; Kawamoto et al., 2015). Yet extant experimental research is limited in explaining which types of real-world relationships become more or less salient in the moments following exclusion.

Understanding who individuals seek out after social exclusion is also increasingly important due to emergence of online technologies. Instead of chatting with a nearby coworker or stranger, people can now message their wisest or kindest friend at almost any moment, including periods of social stress or threat (Holtzman et al., 2017). Hence, technologies that enhance the availability of others allow people to choose between a wider set of recipients in daily life (Trieu et al., 2019). Moreover, research suggests that contextual and emotional factors can shape the way people engage with their social networks, with a substantial portion of social support mobilization being spontaneous or incidental (Smith et al., 2012; Small and Sukhu, 2016). Nonetheless, it is unclear how people *choose* particular ties after an experience of exclusion.

One common way of reaching out to friends and family is through online news sharing, and according to word-of-mouth research, people share more news articles when in high-arousal states (Berger, 2011; Berger and Milkman, 2012). Research on the social sharing of emotions demonstrates that people generally share emotional events with intimate ties (c.f., Rimé, 2009), though the type of close tie chosen (e.g., family vs. partner) varies by age group. However, it is unknown whether negative high-arousal states, such as feelings of exclusion, prompt certain types of relationships to become more or less salient. In this way, the emotional effects of exclusion may shift the personal ties who come into focus, thus changing a form of “social scope” (Kobayashi and Boase, 2014).

In contrast to the emotion sharing literature, here we consider how emotional events can alter personal network scope – i.e., who comes to mind – during subsequent social behavior. Consequently, we tested whether being excluded influences the rate of sharing news articles to personal ties in an unrelated online task, while also assessing changes in which relationships (e.g., close family, weak friends) are preferred. That is, we examined whether social exclusion indirectly redirects attention toward some types of relationships and away from others. In doing so, this study extends past research on social exclusion, word-of-mouth, and social scope in concert.

## Materials and Methods

Ninety-six college students (63 females; aged 18–24) participated in exchange for course credit^1^. The study was conducted over two appointments. In Appointment 1, participants provided information about their personal relationships in their everyday communication network. Participants entered up to 20 family members, 20 calling partners, and 20 texting partners. For calling and texting partners, participants identified their recent contacts from their phones. Given the established role of tie strength in word-of-mouth sharing (Dubois et al., 2016), we collected two measures for each relationship: perceived “closeness” of each contact ranging from (1) do not know to (7) very close and whether participants had seen each contact face-to-face and (within the last week, month, year, or over a year). After a minimum of 5 days, participants came back for Appointment 2 in which they completed two ostensibly unrelated social tasks: *Cyberball* (social exclusion task) and pilot-testing a news website (online sharing task).

### Social Exclusion Task

Cyberball is an exclusion paradigm in which participants complete “a mental visualization task” (Williams et al., 2000; c.f., Dvir et al., 2019), reliably eliciting distress both online and offline (Schneider et al., 2017). In the game, an avatar representing the participant throws a ball with two other avatars. Participants were told they were engaging in the task with two students from nearby colleges. Participants were randomly assigned to one of two conditions. In the inclusion condition, the other avatars were pre-programmed to throw the ball to the participant at regular occasions; in the exclusion condition, the other avatars initially threw the ball to the participant, but later only threw the ball to one another, excluding the participant. Afterward, participants completed a manipulation check, the 20-item Need Threat Scale (NTS; van Beest and Williams, 2006). Responses were assessed on a 7-point scale ranging from 1 (*strongly disagree*) to 7 (*strongly agree*). Higher scores on the NTS indicate greater need *satisfaction*, or less self-reported distress following the experimental manipulation.

### Online Sharing Task

The second task involved “pilot testing” a website for reading and sharing news articles. On the website, participants were asked to read pre-selected news articles. The custom site allowed participants to choose a topic relevant to them (health, sports, science, or technology). Importantly, the side panel of the website provided the opportunity for participants to share articles with friends entered in Appointment 1. Each participant evaluated six different news articles during the task, and the same selection of articles were counterbalanced across conditions. Next to each news article, the site presented four contacts selected randomly from the participants’ own network – two close ties and two weak ties – with whom participants could share the article. The site also included a search option in which participants could share with additional friends from their complete network. Participants were asked to share articles as they normally would in “real life” in order to provide feedback on the best and worst features of the website, but no specific requirements or guidelines for sharing news articles were given.

## Results

To check the effectiveness of the Cyberball manipulation, we computed indices of the belongingness (*α* = 0.76), self-esteem (*α* = 0.69), meaningfulness (*α* = 0.69), and control (*α* = 0.73) sub-scales from the Need Threat Scale^2^. Between-groups one-way ANOVAs were run, which confirmed that Cyberball effectively manipulated social exclusion. Excluded participants felt less included [*M*_included_ = 3.72, *M*_excluded_ = 2.78; *F*(1, 83) = 34.35, *p* < 0.001], lower in self-esteem [*M*_included_ = 2.91, *M*_excluded_ = 2.45; *F*(1, 83) = 9.44, *p* = 0.003], less meaningful [*M*_included_ = 3.52, *M*_excluded_ = 2.80; *F*(1, 83) = 23.92, *p* < 0.001], and less control [*M*_included_ = 3.01, *M*_excluded_ = 2.21; *F*(1, 83) = 21.74, *p* < 0.001].

Next, we identified whether the targets of article sharing were socially distant (closeness = 2–4) or socially close to the participant (closeness = 5–7). Since network cognition differs as a function of whether ties are family members (Brashears, 2013), we also delineated ties as family vs. friends (i.e., non-family ties). Separate analyses were run for number of articles shared with close family, close friends, weak family, and weak friends as outcome variables. We also computed the number of different channels that participants communicated with each of their contacts (i.e., multiplexity; two = both calling and texting; one = calling or texting; zero = neither), and whether the contact had last been interacted with face-to-face. We conducted an analysis of covariance (ANCOVA) assessing the effect of exclusion on number of articles shared with each target type while controlling for individual differences in the amount of overall sharing^3^.

**Figure 1:**
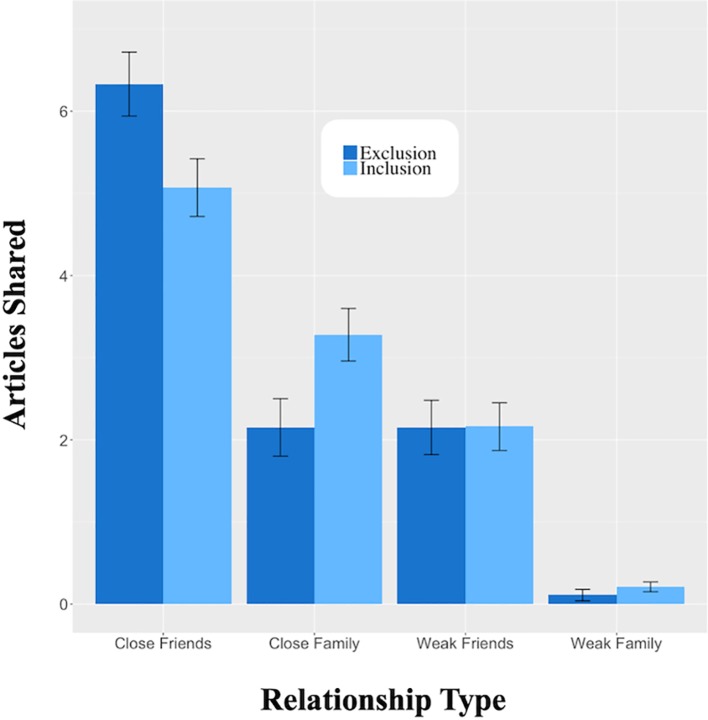
Effect of Cyberball on subsequent news article sharing with friends and family members. Friends and family were defined as either close or weak ties based on the self-reported closeness of the specific relationship. As compared to the inclusion (light), exclusion (dark) increases sharing with close friends and decreases sharing with close family. However, exclusion did not influence sharing with weak friends or family, which remained at lower levels regardless of the manipulation.

All models (described below) evaluating the effects of exclusion on sharing were ANCOVAs. We first evaluated the effect of exclusion on overall sharing but found no significant effect (*F* < 1). However, exclusion drove sharing with different types of targets. Excluded participants shared more articles with close friends (*M* = 6.33 articles, SE = 0.39 articles) than included participants (*M* = 5.07 articles, SE = 0.35 articles), *F*(1, 93) = 5.62, *p* = 0.020, *r* = 0.24 for the effect of exclusion. Additionally, excluded participants also shared fewer articles with close family ties (*M* = 2.13 articles, SE = 0.35 articles) than included participants (*M* = 3.28 articles, SE = 0.32 articles), *F*(1, 93) = 5.64, *p* = 0.020, *r* = 0.24 for the effect of exclusion. Sharing with weak friends and family was unaffected by exclusion, *F* < 1 and *F*(1, 93) = 1.03, *p* = 0.311, respectively. Thus, exclusion increases sharing with close friends. See Figure 1.

**Figure 2:**
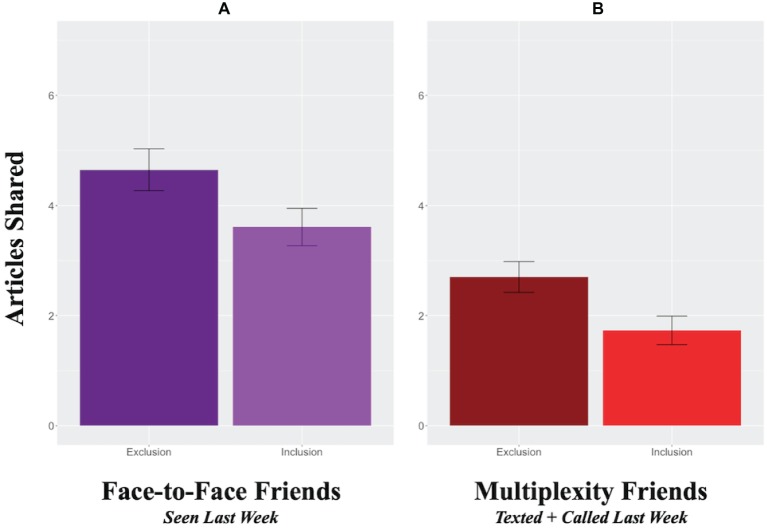
Convergent validity for the primary finding (Figure 1) by examining two additional operationalizations of close ties. As shown in the left panel **(A)**, exclusion (dark) prompted more sharing to friends seen face-to-face in the prior week, as compared to inclusion (light). Similarly, as shown in the right panel **(B)**, excluded (vs. included) participants shared more news articles with friends who they had both texted and called in the previous week (i.e., media multiplexity friendships).

Recent face-to-face interactions are more emblematic of close relationships (Pollet et al., 2011). If exclusion increases sharing with close friends, it should similarly increase sharing with friends participants had physically interacted with recently. As shown in Figure 2A, this was the case: excluded participants shared more articles with friends with whom they had seen face-to-face within the last week (*M* = 4.65 articles, SE = 0.38 articles) than included participants (*M* = 3.61 articles, SE = 0.34 articles), *F*(1, 93) = 4.14, *p* = 0.045, *r* = 0.21. Notably, exclusion did not affect sharing with friends seen face-to-face over longer time scales, including within the month, *F* < 1, or within the year, *F*(1, 93) = 3.24, *p* = 0.075, *r* = 0.18. Exclusion also significantly decreased sharing with friends last seen face-to-face over a year ago, *F*(1, 93) = 8.15, *p* = 0.005, *r* = 0.28. By contrast, exclusion had no effect on sharing with family ties seen face-to-face within the week, *F*(1, 93) = 1.40, *p* = 0.239, within the year, *F* < 1, or over a year ago, *F*(1, 93) = 1.53, *p* = 0.219. However, exclusion decreased sharing with family ties seen in the last month, *F*(1, 93) = 8.68, *p* = 0.004, *r* = 0.29.

Close ties also exhibit media multiplexity; i.e., they are contacted through more communication channels (Haythornthwaite, 2005). If exclusion increases sharing with close friends, it should increase sharing with more multiplex friends. As shown in Figure 2B, this was observed: excluded participants shared more articles with multiplex friends (*M* = 2.70 articles, SE = 0.28 articles) than included participants (*M* = 1.73 articles, SE = 0.26 articles), *F*(1, 93) = 6.43, *p* = 0.013, *r* = 0.25. However, exclusion did not affect sharing with one or zero channel friends, (*F* < 1). In line with the previous sets of analyses, excluded participants shared fewer articles with multiplex family ties *F*(1, 93) = 4.48, *p* = 0.037, *r* = 0.21, but did not share more or less with family ties contacted through one channel, *F*(1, 93) = 1.53, *p* = 0.220, or those contacted through zero channels, *F* < 1.

## Discussion

Which ties are preferred in the moments after exclusion? Our data indicate that close friends are prioritized. Specifically, we find that exclusion increases online news sharing to close friends, but not weak friends or family. These data are consistent with previous studies indicating that elevated arousal can influence unrelated news sharing (e.g., Berger, 2011), and with the large literature showing that exclusion causes people to work to regain acceptance from others who did not perpetrate the exclusion. Our data also extend prior findings by showing that levels of sharing differ according to the type of tie in question. When belongingness is threatened, strong friendships may come to mind as the fastest and safest remedy – and perhaps most worthy of bolstering.

From a more fine-grained standpoint, this study provides initial evidence for the reallocation of network scope. Excluded participants shared more with close friends – and less with close family – across three measures of tie strength: emotional closeness, face-to-face recency, and media multiplexity. Past research shows that family ties are perceived in a fundamentally different way than non-family ties (Brashears and Quintane, 2015). Due to their special status, the results suggest that participants may have shifted priorities, allocating less attention to family members. If family ties are secure by default, draw from a separate pool of belongingness, and do not cause the exclusion, then network focus may adjust to match present goals (e.g., restoring belongingness to a less secure group). Another possibility is that excluded participants avoided weaker ties when sharing due to their similarity to the Cyberball perpetrators (students from nearby colleges). More work is needed to investigate how everyday social experiences shape *in vivo* personal network scope, as well as influence social network characteristics over time (Bayer et al., 2018).

In parallel, our study builds on past work by showing that a negatively arousing *social* activity has the potential shift social scope and transmission. This distinction is significant given that prior research has focused on positive or neutral arousal states, and these manipulations have primarily been induced in non-social ways. Indeed, socially derived emotions may have different carryover effects given the inherently social nature of sharing. At the same time, whereas previous studies found a categorical positive effect of arousal on sharing, we found a more contextual effect based around the type of personal tie. These nuanced effects affirm importance of identifying the boundary conditions of social transmission effects, revealing how subtle changes in word-of-mouth can occur discreetly in the backdrop of daily life.

The observed redirection in social scope also demonstrates the need to reconsider how online technologies are rewiring social transmissions. For instance, this effect warrants comparison to the tele-cocooning hypothesis, which states that use of mobile technologies will strengthen strong ties *at the expense of* weak ties (Kobayashi and Boase, 2014). Although research has established that mobile availability results in people communicating mostly with their core ties (and sometimes feeling closer to them), there is mixed empirical support for tele-cocooning (Campbell, 2015). In the current case, the increased sharing for close friends indicates that exclusion can shift the specific outlets for sharing, as opposed to changing the aggregate level of social closeness or support. As such, our study suggests that future research should reconsider how online availability may influence social network cognition – in context – rather than overall social resources.

Past ostracism research has consistently shown that being excluded prompts subsequent efforts to connect, but largely studied reconnection with generic others. In a similar vein, prior research on personal relationships has often neglected the role of social networks (Parks, 2011), yet how people choose among their online ties is increasingly central to satisfying social needs (Hall and Davis, 2016). Our results show how network availability can tweak the mental equation. By providing the option to share with personal ties, we provide a more naturalistic test on the residual effects of being excluded today. Concurrently, a number of limitations in our study deserve attention to best guide future research. First, our findings related to particular types of relationships are likely to be influenced by the characteristics of our sample (i.e., female college students; young adults). Likewise, the sample was collected at a large university in the midwestern United States, which could affect the types of social networks activated since different relationships are more salient across development (Rimé, 2009); for example, family members may be less prominent within college students’ everyday social networks. Finally, our design used a customized online network generator that synced with a novel news website, which resulted in a sizable share of missing data due to technical glitches. Altogether, researchers should pursue more generalizable samples and replicate these findings through other social network paradigms.

Our study offers initial evidence that daily challenges, when paired with online availability, may shift communication in incidental ways. We find convergent evidence that the experience of exclusion increases sharing with close friends, and decreases sharing with close family. Although we initially hypothesized a main effect of exclusion on sharing, these findings highlight a more nuanced effect on the specific outlets for sharing (vs. total amount). This result can be explored with future research, while also attending to the implications for both discrete ties and the overall structure of personal networks. Future studies are thus needed to clarify how social exclusion shapes personal network scope, and how those cognitive mechanisms relate to social network structure over time.

## Data Availability

The data that support the findings of this study are openly available on OSF at: https://osf.io/utaqn/?view_only=d283da6421b34c55b8c10ebe8efa722d.

## Ethics Statement

This study was carried out in accordance with the recommendations of University of Michigan Institutional Review Board (IRB) with written informed consent from all subjects. All subjects gave written informed consent in accordance with the Declaration of Helsinki. The protocol was approved by the University of Michigan IRB.

## Author Contributions

JB, DH, and EF wrote the main manuscript. JB and DH conducted the analyses. All authors assisted in the study design, data collection, and manuscript preparation.

### Conflict of Interest Statement

The authors declare that the research was conducted in the absence of any commercial or financial relationships that could be construed as a potential conflict of interest.

## Footnotes

^1^One hundred and twenty-eight students attended both appointments, but the data of thirty-two participants were lost due to technical glitches in our customized social network and news website procedure.

^2^Eleven cases were missing NTS data, bringing the total number of cases for this analysis down to 85.

^3^Effects did not differ by whether contacts were selected from the side panel or searched.
